# In clinically hyperthyroid cats, is I-131 treatment superior to thyroidectomy in normalising serum T4 level?

**DOI:** 10.18849/ve.v7i4.433

**Published:** 2022-12-16

**Authors:** Alexander Davies+

**Keywords:** FELINE, HYPERTHYROIDISM, I-131, RADIOACTIVE IODINE, RADIOIODINE, T4, THRYOIDECTOMY, THYROXINE

## Abstract

PICO question

In clinically hyperthyroid cats, is iodine-131 (I-131) treatment superior to unilateral or bilateral thyroidectomy in normalising serum thyroxine (T4) levels?

Clinical bottom line

Category of research question

Treatment.

The number and type of study designs reviewed

35 papers were critically reviewed. These were mostly retrospective studies with a small proportion of prospective cohort studies.

Strength of evidence

Moderate.

Outcomes reported

More papers were available evaluating the effect of radioiodine therapy on T4 levels compared to thyroidectomy. Long-term follow-up of T4 is a relatively new component of study designs. Most papers suggested between 40–87% cats had normal T4 6 months after treatment. 19–47% cats receiving unilateral or bilateral thyroidectomy, and 100% cats receiving radioiodine therapy were in long-term remission in one study.

Conclusion

In view of the evidence and outcomes from the studies, there is moderate evidence that I-131 treatment is superior to unilateral or bilateral thyroidectomy.

How to apply this evidence in practice

The application of evidence into practice should take into account multiple factors, not limited to: individual clinical expertise, patient’s circumstances and owners’ values, country, location or clinic where you work, the individual case in front of you, the availability of therapies and resources.

Knowledge Summaries are a resource to help reinforce or inform decision making. They do not override the responsibility or judgement of the practitioner to do what is best for the animal in their care.

## Clinical scenario

On diagnosing feline hyperthyroidism, clinicians are quick to offer the therapeutic options including definitive treatments (radioactive iodine treatment or surgery) or lifelong management (antithyroid drugs or iodine- restricted diets). These options can pose more questions than solutions for many owners, who wish to provide a simple and noninvasive fix for their typically geriatric pet. For owners pursuing a definitive cure it can be difficult to weigh up whether radioactive iodine treatment or thyroidectomy is best. Radioactive iodine is only available at limited centres and some owners are unhappy with being separated from their cat for an extended period of time. Surgery is invasive, irreversible and has an added anaesthetic risk for geriatric or co-morbid cats. The cost of these therapeutic options is an important factor that owners must weigh up, deciding between surgery which is performed in-house in many primary care centres versus referral for radioiodine treatment. Is radioactive iodine really the safest and best option to help manage the disease?

## The evidence

35 papers were evaluated exploring how radioiodine treatment reduced T4 levels to within reference range in cats with confirmed hyperthyroidism (clinical signs, elevated T4 levels ± thyroid imaging). The majority of these were retrospective studies using a cohort formula that compared thyroid parameters amongst other measures before and after radioiodine treatment. A total of five papers were evaluated that looked at how thyroidectomy (either unilateral or bilateral) reduced T4 levels to within normal reference range.

## Summary of the evidence

### Studies that included cats that underwent thyroidectomy

**Birchard et al. (1984):** 

Population:	Client-owned cats with confirmed hyperthyroidism that underwent either unilateral or bilateral thyroidectomy.
Sample size:	85 cats.
Intervention details:	Cats were treated with either unilateral or bilateral thyroidectomy.
Study design:	Cohort study.
Outcome Studied:	Serum T4 concentration.
Main Findings (relevant to PICO question):	32 cats had unilateral thyroidectomy and 53 had bilateral thyroidectomy, based on a thyroid scan. All cats had serum thyroid hormone concentrations at normal or below the normal reference interval within 48 hours of surgery. Most cats with bilateral thyroidectomy were treated postoperatively with oral thyroxine supplementation. This was discontinued in most cats within 3–6 months with no evidence of clinical or laboratory hypothyroidism. Relapse of hyperthyroidism occurred in four cats, 8–44 months following bilateral thyroidectomy, confirmed with a repeated thyroid imaging. Most cats with unilateral thyroidectomy had T4 levels below normal range for 2–3 months after surgery but did not require thyroid supplementation.
Limitations:	Cats with euthyroidism or hypothyroidism were not separated in analysis. Subclinical hypothyroidism not determined using thyroid stimulating hormone (TSH) measurements. Only cats with clinical disease were followed-up with long-term sampling. Cats did not receive a wash out period for antithyroid drugsprior to surgery. Referral population.

**Graves et al. (1994):** 

Population:	Client-owned cats with confirmed hyperthyroidism referred for bilateral thyroidectomy and healthy control cats.
Sample size:	13 cats.
Intervention details:	Cats with hyperthyroidism underwent bilateral thyroidectomy (n = 13).
Study design:	Cohort study.
Outcome Studied:	Outcome of bilateral thyroidectomy before and 30 days after: Serum thyroxine (T4) concentration. Complete blood count (CBC), biochemistry, urinalysis. Glomerular filtration rate (GFR).
Main Findings (relevant to PICO question):	30 days post-surgery, mean serum T4 was reduced by 90%. T4 fell to with the reference interval (10–48 nmol/L) in 6/13 (46%) hyperthyroid cats 30 days after surgery. T4 fell below the reference interval in 7/13 (54%) hyperthyroid cats 30 days after surgery. All cats had a resolution of clinical signs and gained weight 30 days after bilateral thyroidectomy. No significant difference in T4 was found in control cats at day 0 and day 30.
Limitations:	Relatively small sample sizes in each group. Subclinical hypothyroidism not determined (no thyroid stimulating hormone (TSH) measurements). Follow-up was only at 30 days and no longer term T4 evaluation was performed.

**Naan et al. (2006):** 

Population:	Client-owned cats with confirmed hyperthyroidism (elevated thyroxine [T4], clinical signs and thyroid scintigraphy) referred for thyroidectomy (unilateral and bilateral depending on imaging) between 1998 and 2002.
Sample size:	101 cats.
Intervention details:	Cats were treated with either bilateral or unilateral thyroidectomy using the modified intracapsular dissection technique.
Study design:	Retrospective case-control study.
Outcome Studied:	Outcome determined by telephone interview with owners or referring veterinarians (median 13 months after surgery): Resolution or recurrence of clinical signs. Plasma T4 levels if measured.
Main Findings (relevant to PICO question):	95 cats received their first thyroidectomy surgeries at the Utrecht University Clinic by the same surgeon. 81 cats received bilateral thyroidectomy with all cats experiencing remission of their hyperthyroidism. Five cats had bilateral thyroidectomy and removal of additional ectopic tissue. 57/95 (60%) cats experienced remission at follow-up and 38/95 (40%) had recurrence of disease. Recurrence of hyperthyroidism after surgery was significantly higher in cats with ectopic hyperplastic thyroid tissue. Surgeon experience may be an important factor in determining the long-term outcome of thyroidectomy, especially with regards to postoperative hypocalcaemia. Nine cats had unilateral thyroidectomy and all had remission of disease at follow-up.
Limitations:	Referral population. Follow-up checks were through interview and although some were with referral veterinarians, the follow-up data may be more subjective than objective. Remission was defined by interview mainly by resolution of clinical signs. T4 levels were only measured where available. The possibility of cats with subclinical disease cannot be excluded. Retrospective studies are relatively low down on the evidence hierarchy.

**Covey et al. (2019):** 

Population:	Client-owned cats with confirmed hyperthyroidism that had undergone bilateral thyroidectomy.
Sample size:	Cross-sectional study (n = 68).Longitudinal study (n = 23).
Intervention details:	Cats with confirmed hyperthyroidism treated with bilateral thyroidectomy.
Study design:	Retrospective cross-sectional and longitudinal study.
Outcome Studied:	Objective assessment of: Bodyweight. Total thyroxine (TT4) concentration: Hypothyroidism assessed as TT4 < 10 nmol/L. Euthyroidism assessed as TT4 10–55 nmol/L. Hyperthyroidism assessed as TT4 > 55 nmol/L. Thyroid stimulating hormone (TSH) concentration. Urine specific gravity (USG). Creatinine concentration. Symmetric dimethylarginine (SDMA) concentration. Subjective assessment of: Body condition score (BCS).
Main Findings (relevant to PICO question):	Cross-sectional study (n = 68): 13/68 (19%) cats were euthyroid 6 months post- surgery. 15/68 (22%) cats had persistent or recurrent hyperthyroidism 6 months post-surgery. 33/68 (49%) cats were hypothyroid 6 months post- surgery. Overt hypothyroidism 6 months post-surgery was greater in cats that had both thyroid glands removed in one procedure 46/68 (67.9%) compared to having staged thyroidectomies 27/68 (40%). Longitudinal study 595–1955 days post-surgery (n = 23): 4/23 (17%) cats remained hypothyroid. 19/23 (83%) cats were euthyroid transiently. 10/23 (44%) cats developed recurrent hyperthyroidism. Cats have changes in thyroid function long-term following bilateral thyroidectomy with a high incidence of recurrent hyperthyroidism. Main findings: A high proportion of cats develop recurrent hyperthyroidism following bilateral thyroidectomy. Long-term after surgery, cats remain euthyroid, or progress to hyperthyroidism over years.
Limitations:	Retrospective studies are relatively low down on the evidence hierarchy. Samples used had been in storage for many years (72/151 samples stored for 10 years or more at 80 °C) with numerous freeze-thaw cycles. Total T4 was only followed-up longer term on a small proportion of cats. Medical stabilisation was attempted on 60/68 (88%) cats prior to surgery with varying success. Total T4 was only measured in 45 cats prior to surgery showing that only 24 cats had controlled thyroxine (T4) levels prior to surgery. Subclinical hypothyroidism not determined (no thyroid stimulating hormone [TSH] measurements). Referral population.

### Studies that included cats that underwent thyroidectomy and radioiodine treatment

**DiBartola et al. (1996):** 

Population:	Client-owned cats with confirmed hyperthyroidism (clinical signs, palpable goitre or high serum thyroxine [T4] concentration) randomly treated with radioiodine therapy, bilateral thyroidectomy or methimazole.
Sample size:	58 cats.
Intervention details:	27 cats were treated with radioiodine (2.1–7.2 mCi, equivalent to 78–267 MBq). 22 cats received a bilateral thyroidectomy. Nine cats were medically managed with methimazole.
Study design:	Clinical trial.
Outcome Studied:	Outcome of radioiodine therapy measured before, 30 days after and 90 days after: Serum T4 concentration. Renal parameters – serum creatinine, blood urea nitrogen (BUN), urine specific gravity (USG).
Main Findings (relevant to PICO question):	Radioiodine group (n = 27): Pretreatment T4 = 11.0 ug/dl (141.6 nmol/L). Post-treatment T4 30 days = 1.6 ug/dl (20.6 nmol/L). Post-treatment T4 90 days = 2.0 ug/dl (25.7 nmol/L). Serum T4 concentrations were significantly lower following radioiodine treatment (P < 0.001) but there was no significant difference between day 30 and day 90 post-treatment. Bilateral thyroidectomy group (n = 22): Pretreatment T4 = 9.3 ug/dl (119.7 nmol/L). Post-treatment T4 30 days = 2.6 ug/dl (33.5 nmol/L). Post-treatment T4 90 days = 2.2 ug/dl (28.3 nmol/L). Serum T4 concentrations were significantly lower following bilateral thyroidectomy (P < 0.001) but there was no significant difference between day 30 and day 90 post-treatment. No significant difference was reported between the radioiodine and thyroidectomy groups. The nine cats in the methimazole group were not commented on as there were separate to the PICO question of this summary.
Limitations:	The reference range of T4 was not reported for laboratories used across different private veterinary practices. Relatively small sample sizes in each group. Radioiodine dose was calculated based on clinical signs rather than uptake studies. Subclinical hypothyroidism not determined (no thyroid stimulating hormone [TSH] measurements).

### Studies that included cats that underwent radioiodine treatment

**Turrel et al. (1984):** 

Population:	Client-owned cats with confirmed hyperthyroidism (clinical signs, elevated thyroxine (T4) levels, thyroid scans) referred for radioiodine therapy.
Sample size:	11 cats.
Intervention details:	Cats were treated with intravenous radioiodine (1.0–5.9 mCi, equivalent to 37–219 MBq).
Study design:	Retrospective cohort study.
Outcome Studied:	Total T4 concentration and technetium 99 m thyroid scans were performed before treatment and at 2–3 month intervals after for a total of 6–18 months.
Main Findings (relevant to PICO question):	7/11 (63.6%) cats were clinically and serologically (T4 levels) euthyroid after radioiodine therapy. Six cats were euthyroid after one dose of radioiodine and one cat required a second dose after 6 months. 2/11 (18.1%) cats had a partial response to radioiodine therapy where T4 levels dropped but cats remained clinically hyperthyroid. 2/11 (18.1%) cats were hypothyroid 1 month and 11 months, respectively, after radioiodine therapy.
Limitations:	Referral population. Four cats did not receive a radioiodine kinetic tracer study to calculate their radioiodine dose so they could have been under- or over-dosed. Retrospective data is a weaker form of evidence. Small sample size that may be unrepresentative of the general hyperthyroid cat population. Subclinical hypothyroidism not determined (no thyroid stimulating hormone [TSH] measurements).

**Meric et al. (1986):** 

Population:	Client-owned cats with confirmed hyperthyroidism referred for radioiodine therapy.
Sample size:	31 cats.
Intervention details:	Cats were treated with radioiodine (1.5–6.13 mCi, equivalent to 56–227 MBq).
Study design:	Prospective cohort study.
Outcome Studied:	Pre- and post-radioiodine treatment (12–48 hour intervals until discharge) measurement of: Serum thyroxine (T4) levels. Re-evaluation of serum T4 levels 1 months after radioiodine therapy.
Main Findings (relevant to PICO question):	16/31 (51.6%) cats had T4 within the normal reference interval by post-treatment day 4. 21/31 (67.7%) cats had T4 within the normal reference interval by post-treatment day 8. 9/31 (29%) cats had decreased T4 levels but were still above the normal reference interval. One cat had T4 levels below the normal reference range but did not show clinical signs of hypothyroidism.
Limitations:	Fairly small population size. Referral population. Follow-up was only performed for 1 month.

**Meric & Rubin (1990):** 

Population:	Client-owned cats with confirmed hyperthyroidism referred for radioiodine therapy.
Sample size:	60 cats.
Intervention details:	Cats were treated with radioiodine (4 mCi equivalent to 148 MBq).
Study design:	Retrospective cohort study.
Outcome Studied:	Pre- and post-radioiodine treatment (30–850 days; average 204 days) measurement of: Serum thyroxine (T4) levels.
Main Findings (relevant to PICO question):	50/60 (83%) cats had T4 levels within the normal reference interval at re-evaluation. 5/60 (8%) were hypothyroid at re-evaluation, without clinical signs. Three of these cats became euthyroid by 6 months post-treatment. 5/60 (8%) remained hyperthyroid after treatment, although T4 had significantly reduced post-therapy.
Limitations:	Referral population. Subclinical hypothyroidism not determined (no thyroid stimulating hormone [TSH] measurements). Follow-up was only performed for 1 month.

**Jones et al. (1991):** 

Population:	Client-owned cats with confirmed hyperthyroidism (clinical signs, increased serum thyroxine [T4]) referred for radioiodine therapy.
Sample size:	32 cats.
Intervention details:	Cats were treated with radioiodine (1.1–2.7 mCi, equivalent to 41–100 MBq).
Study design:	Cohort study.
Outcome Studied:	Outcome of radioiodine therapy measured after 3–4 months: Total and free T4 concentration.
Main Findings (relevant to PICO question):	28/32 (87.5%) cats were euthyroid following radioiodine. 3/32 (9%) cats remained hyperthyroid after radioiodine One cat became hypothyroid after radioiodine treatment. This cat had received bilateral thyroidectomy and a recurrence of hyperthyroid clinical signs 18 months prior to radioiodine. The 25 cats surviving remained euthyroid for 1–3 years from the initial radioiodine treatment.
Limitations:	Small population size. Radioiodine dose was calculated based on clinical signs rather than uptake studies. Subclinical hypothyroidism not determined (no thyroid stimulating hormone [TSH] measurements). A referral population may not represent the general feline population since many cats are only referred due to a difficulty controlling their hyperthyroidism, suggesting more severe disease. Particularly evident in this study since one cat was receiving radioiodine due to a relapse of hyperthyroidism after bilateral thyroidectomy.

**Malik et al. (1993):** 

Population:	Client-owned cats with confirmed hyperthyroidism referred for radioiodine therapy.
Sample size:	40 cats.
Intervention details:	Cats were treated with a single dose of oral radioiodine (200–300 MBq).
Study design:	Case series.
Outcome Studied:	Total plasma thyroxine (T4) concentration pre-radioiodine and 4–23 days after treatment (mainly 10 days after dosing). Resolution of clinical signs.
Main Findings (relevant to PICO question):	36/40 (90%) cats had a resolution of clinical signs and low or normal T4 after radioiodine treatment. The remainder of cats were persistently hyperthyroid.
Limitations:	Euthyroid or hypothyroid cats were not separated. No exclusion of non-thyroidal illness or concurrent disease was made. Pre-radioiodine antithyroid medication was not considered. Referral population.

**Mooney (1994):** 

Population:	Client-owned cats with confirmed hyperthyroidism referred for radioiodine therapy.
Sample size:	50 cats.
Intervention details:	Cats were treated with radioiodine (80–00 MBq). Radioiodine was injected intravenously in 27 cats and injected subcutaneously in 23 cats.
Study design:	Cohort study.
Outcome Studied:	Outcome of radioiodine therapy measured before, 30 days after and in some cats 3–5 months after and 32 months: Serum thyroxine (T4) concentration.
Main Findings (relevant to PICO question):	47/50 (94%) cats became euthyroid following radioiodine treatment. Significant decrease in serum T4 levels from a mean of 181.3 ± 111.4 nmol/L to a mean of 19.0 ± 29.6 nmol/L 30 days after injection of radioiodine. Five cats remained hyperthyroid following treatment but serum T4 levels were decreased compared to pretreatment. Of these cats: Two cats became euthyroid within 3–5 months of therapy. Two cats were lost to follow-up. The remaining cat was treated again after 4 months and become euthyroid. No cats became clinically hypothyroid and serum T4 levels below reference range was only transient. Recurrence of hyperthyroidism did not happen in any cat in follow-up periods up to 32 months. No difference between intravenous or subcutaneous radioiodine injection.
Limitations:	Subclinical hypothyroidism not determined (no thyroid stimulating hormone [TSH] measurements). Referral population. Variable durations between radioiodine and follow-up samples.

**Slater et al. (1994):** 

Population:	Client-owned cats with confirmed hyperthyroidism referred for radioiodine therapy.
Sample size:	236 cats.
Intervention details:	Cats were treated with a single dose of oral radioiodine (2.8–8.9 mCi equivalent to 104–330 MBq).
Study design:	Retrospective case-control study.
Outcome Studied:	The measures were performed 3–18 months following radioiodine treatment. Total plasma thyroxine (T4) concentration. Resolution of clinical signs.
Main Findings (relevant to PICO question):	200/236 (85%) cats were euthyroid after treatment. 10/236 (4%) cats were hyperthyroid after treatment. 22/236 (9%) cats were hypothyroid after treatment.
Limitations:	Retrospective studies are relatively low down on the evidence hierarchy. Multivariate analyses may have been more suitable than bivariate analyses for the data in this study. Variable durations between radioiodine and follow-up samples. Long-term follow-up was often carried out by telephone communications with owners. Response to radioiodine was characterised vaguely. Cats were asymptomatic (normal or low T4 with no clinical signs), hyperthyroid (clinical signs and high T4) or hypothyroid (clinical signs and low T4). This does not account for subclinical hypothyroidism and as such the true number of euthyroid cats cannot be determined. Referral population.

**Théon et al. (1994):** 

Population:	Client-owned cats with confirmed hyperthyroidism referred for radioiodine therapy.
Sample size:	120 cats.
Intervention details:	Cats were treated with intravenous (n = 60) or subcutaneous (n = 60) radioiodine (159 Gy).
Study design:	Randomised controlled trial.
Outcome Studied:	Total T4 concentration was measured before treatment and at 1, 3 and 6 months post-treatment.
Main Findings (relevant to PICO question):	51/60 (85%) cats treated with IV radioiodine were euthyroid 4 years after treatment. 50/60 (83%) cats treated with SC radioiodine were euthyroid 4 years after treatment.
Limitations:	Referral population. Subclinical hypothyroidism not determined (no thyroid stimulating hormone [TSH] measurements).

**Peterson et al. (1995):** 

Population:	Cats with confirmed hyperthyroidism referred for radioiodine treatment.
Sample size:	524 cats.
Intervention details:	Cats were treated with radioiodine (2–6 mCi equivalent to 74–222 MBq). 14 cats had partial thyroidectomy prior to radioiodine treatment.
Study design:	Prospective case series.
Outcome Studied:	Objective measurement of: Total thyroxine (T4) concentration. Samples measured pretreatment and again on day of discharge, 2–3 months, 6–12 months and then yearly after radioiodine treatment.
Main Findings (relevant to PICO question):	At discharge, 448/524 (85.5%) cats had serum T4 concentrations within the reference range. 2–3 months after treatment, serum T4 concentration was within the reference range in 422/519 (81.3%) cats. 6–12 months after treatment, serum T4 concentration was within the reference range in 437/502 (87.1%) cats. Overall, the response to treatment was ‘good’ (resolution of clinical signs and T4 levels within the reference range) in 485/515 (94.2%) longer-term. 11/524 (2.1%) cats developed clinical hypothyroidism. By 6 months post-radioiodine, only 8/524 (1.5%) cats were persistently hyperthyroid.
Limitations:	T4 levels below the lower reference limit determined hypothyroidism without accounting for T4-lowering effect of non-thyroidal diseases. The statistical analysis did not account for the grouping of cats into different groups depending on the severity of hyperthyroidism. Referral population.

**Adams et al. (1997):** 

Population:	Client-owned cats with confirmed hyperthyroidism (increased serum thyroxine (T4) and scintigraphic findings) referred for radioiodine therapy.
Sample size:	22 cats.
Intervention details:	Cats were treated with radioiodine (4.5 mCi equivalent to 167 MBq).
Study design:	Prospective cohort study.
Outcome Studied:	Pre- and post-radioiodine treatment (30 days after) measurement of: Serum total T4 concentration; Biochemistry; Urinalysis.
Main Findings (relevant to PICO question):	T4 was normal in two cats after 30 days. T4 was below normal in 15 cats after 30 days. T4 was above normal in five cats after 30 days. Response to pretreatment antithyroid medication was not recorded that may affect the population characteristics. T4 was normal in 10 cats and above normal in 10 cats that were also evaluated immediately following radioiodine treatment. Extended follow-up data was available for 14 cats. 10/14 cats with below normal T4 at 30 days had normal T4 values long-term. Two cats persisted with low T4 values and two cats persisted with high T4 values. No follow-up data was available for the two cats with normal T4 values on day 30.
Limitations:	Small population size. Long-term follow-up was not performed in all cats. Referral population. Subclinical hypothyroidism not determined (no thyroid stimulating hormone [TSH] measurements).

**Chun et al. (2002):** 

Population:	Client-owned cats with confirmed hyperthyroidism referred for radioiodine therapy.
Sample size:	193 cats.
Intervention details:	Cats were treated with radioiodine (4 mCi equivalent to 148 MBq).
Study design:	Retrospective case-control study.
Outcome Studied:	Thyroxine (T4) concentration. Resolution of clinical signs.
Main Findings (relevant to PICO question):	Quantitative values were not reported but 12 month follow-up measurements of T4 were reported in a histogram. The number of cats with T4 within the normal reference intervalincreased from approximately 100–150 from 1 week to 6 months after radioiodine treatment. This reduced to approximately 125 cats after 12 months. Cats that had elevated T4 levels 1 week after radioiodine treatmentfell to 0 cats at 6 months and 12 months. Cats with T4 levels below the reference interval at 1 week after radioiodine treatment were slightly higher by 12 months post- treatment. The specific values were not reported.
Limitations:	Not all cats were followed-up at every time point. For inclusion, two time points were required out of 1 week and 1, 3, 6 and 12 months post-therapy. Retrospective studies are relatively low down on the evidence hierarchy. Referral population. Subclinical hypothyroidism not determined (no thyroid stimulating hormone [TSH] measurements).

**van Dijl & Hof (2008):** 

Population:	Client-owned cats with confirmed hyperthyroidism referred for radioiodine treatment.
Sample size:	83 cats.
Intervention details:	Cats were treated with radioiodine (4–6 mCi, equivalent to 148–222 MBq).
Study design:	Case series.
Outcome Studied:	Objective measurement of: Total thyroxine (T4) concentration; Urea and creatinine. Samples measured pretreatment, 10 days after and several months after treatment.
Main Findings (relevant to PICO question):	After 10 days and several months, T4 had fallen below the upper reference limit in 64/83 (77%) and 72/83 (87%) cats, respectively. 4/83 (5%) cats T4 levels decreased below the lower reference limit. Two of these cats had clinical signs of hypothyroidism.
Limitations:	The analyses did not account for the effect of pre- radioiodine antithyroid drugs. 65/83 (78%) cats had been pretreated with antithyroid medication with a withdrawal of only 3 days prior to radioiodine treatment. Samples across the study population were analysed in different laboratories with different reference ranges. Referral population.

**Van Hoek et al. (2008):** 

Population:	Client-owned cats with confirmed hyperthyroidism referred for radioiodine therapy.
Sample size:	15 cats.
Intervention details:	Cats were treated with radioiodine (74 MBq).
Study design:	Prospective cohort study.
Outcome Studied:	Pre- and post-radioiodine treatment (1, 4, 12 and 24 weeks after) measurement of: Serum total T4 concentration, Biochemistry, Glomerular filtration rate (GFR).
Main Findings (relevant to PICO question):	13/15 (87%) cats had serum total T4 concentrations within the normal reference range 24 weeks following radioiodine therapy.
Limitations:	Referral population. Small population size. Cats being medically treated prior to radioiodine were not excluded and a wash-out period for medication was not noted. Subclinical hypothyroidism not determined (no thyroid stimulating hormone [TSH] measurements).

**Feeney et al. (2011):** 

Population:	Client-owned cats with confirmed hyperthyroidism referred for radioiodine treatment.
Sample size:	97 cats.
Intervention details:	Cats were treated with radioiodine (3.06–6.04 mCi, equivalent to 114–224 MBq).
Study design:	Retrospective case-control study.
Outcome Studied:	Objective measurements pre- and post-intervention (8–11 weeks after) of: Total thyroxine (T4) concentration; Biochemistry: Aspartate aminotransferase (AST), alanine aminotransferase (ALT), alkaline phosphatase (ALP), total bilirubin, cholesterol, albumin, blood urea nitrogen (BUN), creatinine, phosphorous, calcium; Total protein.
Main Findings (relevant to PICO question):	63/97 (64.9%) cats had normal T4 values in the normal range.
Limitations:	Only one pretreatment and post-treatment sample was taken. Longer-term sampling would be after follow-up to the response to radioiodine treatment. Retrospective studies are relatively low down on the evidence hierarchy. Different laboratories were used to measure T4 across the study group, and as such values were reported as a function of the specified normal range of each laboratory. Referral population. Cats that have adverse reactions to methimazole prior to therapy may response differently to radioiodine to cats that do not have adverse reactions.

**Geesaman et al. (2016):** 

Population:	Client-owned cats with confirmed hyperthyroidism referred for radioiodine therapy.
Sample size:	29 cats.
Intervention details:	Cats were treated with radioiodine (111–186 MBq).
Study design:	Prospective cohort study.
Outcome Studied:	Pre- and post-radioiodine treatment (60 days after) measurement of: Serum total thyroxine (T4) concentration; Blood pressure; Haematocrit (HCT), blood urea nitrogen (BUN), creatinine, urine specific gravity (USG), alanine transaminase (ALT), alkaline phosphatase (ALP), albumin, globulin, TLI, folate, cobalamin, methylmalonic acid (MMA).
Main Findings (relevant to PICO question):	29/39 (74%) cats had total T4 concentrations within the normal reference interval 60 days after radioiodine treatment.
Limitations:	Clinical work-up did not completely rule out all concurrent illness. Small population size. Referral population. Subclinical hypothyroidism not determined (no thyroid stimulating hormone [TSH] measurements). Resolution of clinical signs was also not measured in follow-up samples. Non-thyroidal illness may have played a role in lowering T4 concentrations.

**Volckaert et al. (2016):** 

Population:	Client-owned cats with confirmed hyperthyroidism referred for radioiodine therapy.
Sample size:	167 cats.
Intervention details:	Cats were treated with radioiodine (69.56–372.22 MBq).
Study design:	n = 39 – retrospective case-control study.n = 128 – prospective cohort study.
Outcome Studied:	Pre- and post-radioiodine treatment (6 months after) measurement of: Serum total thyroxine (T4) concentration. Thyroid volume based of scintigraphic findings.
Main Findings (relevant to PICO question):	111/167 (66.5%) cats had a total T4 concentration within the normal reference interval at 6 months after therapy. 40/167 (24%) cats had a total T4 concentration below the normal reference interval at 6 months after therapy. 16/167 (9.5%) cats had a total T4 concentration above the normal reference interval at 6 months after therapy. 62/111 (55.9%) of the euthyroid cats were bilaterally affected, 40/111 (36%) were unilaterally affected and 9/111 (8.1%) showed the presence of ectopic thyroid tissue.
Limitations:	Referral population. Retrospective studies are relatively low down on the evidence hierarchy, although the prospective and retrospective groups were controlled well. Subclinical hypothyroidism not determined (no thyroid stimulating hormone [TSH] measurements). Resolution of clinical signs was also not measured in follow- up samples. Non-thyroidal illness may have played a role in artificially lowering T4 concentrations.

**Lucy et al. (2017):** 

Population:	Client-owned cats with confirmed hyperthyroidism referred for radioiodine treatment.
Sample size:	189 cats.
Intervention details:	Cats were treated with radioiodine (2-4 mCi, equivalent to 74–148 MBq). 39 cats received standard dose iodine-131 (I-131). 150 cats received low-dose I-131.
Study design:	Non-randomised cohort study.
Outcome Studied:	Objective assessment of serum thyroxine (T4) concentration, thyroid stimulating hormone (TSH) and creatinine pre-intervention and 1, 3 and 6 months following radioiodine treatment. Cats were treated with two different I-131 doses (2 mCi or 4 mCi) depending on the severity of hyperthyroidism before treatment. Euthyroidism was determined as T4 0–9 – 9 μg/dL or TSH <0.3 ng/mL.
Main Findings (relevant to PICO question):	Cats treated a standard radioiodine dose (4 mCi) had significantly lower serum T4 concentrations and higher TSH at 1, 3 and 6 months, compared with cats treated with lower dose radioiodine (2 mCi). Of the cats receiving standard dose (4 mCi) treatment (17/39 were euthyroid after 6 months (44%). Of the cats receiving low dose (2 mCi) treatment 113/150 were euthyroid after 6 months (75%). Cats treated with the standard dose (4 mCi) were significantly less likely to be euthyroid than those treated with the low dose (2 mCi). Cats treated with the lower dose (2 mCi) had lower serum creatinine concentrations and were less likely to develop iatrogenic hypothyroidism.
Limitations:	The number of animals in each dose group was different that may hide the real effect of dose on radioiodine result. Allocation of each dose was not randomised and cats were differed only by the hospital and hospital protocol they were referred to, introducing potential bias. Different laboratories were used for pre-intervention and follow-up samples across the study population. Referral population.

**Marsilio et al. (2017):** 

Population:	Client-owned cats with confirmed hyperthyroidism referred for radioiodine therapy.
Sample size:	42 cats.
Intervention details:	Cats were treated with radioiodine (200–300 MBq).
Study design:	Prospective cohort study.
Outcome Studied:	Pre- and post-radioiodine treatment (4 weeks) measurement of: Body condition. Leptin and ghrelin concentration. Serum total thyroxine (T4) concentration.
Main Findings (relevant to PICO question):	10/42 (24%) cats were euthyroid 4 weeks after radioiodine treatment. 28/42 (67%) cats were hypothyroid 4 weeks after radioiodine treatment. 4/42 (9%) cats were hyperthyroid 4 weeks after radioiodine treatment.
Limitations:	Fairly small population size. Referral population.

**Stock et al. (2017):** 

Population:	Client-owned cats with confirmed hyperthyroidism referred for radioiodine therapy.
Sample size:	42 cats.
Intervention details:	Cats were treated with radioiodine (67.71–455.10 MBq depending on hyperthyroidism severity based on clinical signs, serum total thyroxine (TT4) and thyroid-salivary gland ratio on scintigraphy).
Study design:	Prospective cohort study.
Outcome Studied:	Pre- and post-radioiodine treatment (after 1 month) measurement of: Physical examination. CBC and serum biochemistry. Serum total thyroxine (T4) concentration. Urinalysis: urine specific gravity (USG), urinary pH, urine protein creatinine ratio, sediment examination and bacterial culture).
Main Findings (relevant to PICO question):	27/42 (64%) cats had a T4 concentration within the normal reference interval 1 month after radioiodine treatment. 15/42 (36%) cats had a T4 concentration below the normal reference interval 1 month after radioiodine treatment.
Limitations:	Subclinical hypothyroidism not determined (no thyroid stimulating hormone [TSH] measurements). The wash out period for antithyroid drugs prior to radioiodine treatment was only 10 days. Small population size. Response to radioiodine was only measured 1 month after. A longer-term follow-up may identify how radioiodine normalises T4 concentration, or not. Referral population.

**Vagney et al. (2017):** 

Population:	Client-owned cats with confirmed hyperthyroidism referred for radioiodine treatment.
Sample size:	96 cats.
Intervention details:	Cats were treated with radioiodine (169 MBq).
Study design:	Retrospective case-control study.
Outcome Studied:	Objective assessment of parameters at two follow-up times after radioiodine treatment: Serum total thyroxine (TT4) concentration.
Main Findings (relevant to PICO question):	Median serum TT4 concentration significantly reduced at first and second follow-ups compared to diagnosis. Two cats developed iatrogenic hypothyroidism. Two cats had persistent hyperthyroidism. Two cats relapsed into hyperthyroidism 1.6 and 3.4 years after radioiodine.
Limitations:	Different laboratories measured T4 concentrations across the study population. Although ratios were calculated to reduce the impact of this, a better control would be to use one standardised laboratory throughout the study. Ratio-corrected thyroid values cannot be directly compared with other studies and do not stipulate true euthyroidism. Subclinical hypothyroidism not determined (no thyroid stimulating hormone [TSH] measurements). Retrospective studies are relatively lower down on the evidence hierarchy. Referral population.

**Morré et al. (2018):** 

Population:	Client-owned cats with confirmed hyperthyroidism referred for radioiodine therapy.
Sample size:	57 cats: Control group using standard dose (n = 23). Treatment group based of scintigraphic findings (n = 34).
Intervention details:	Cats (n = 34) were treated with intravenous or subcutaneous radioiodine (3.0, 3.5 or 4.5 mCi variable based on scintigraphic findings, equivalent to 111, 130 or 167 MBq). Cats (n = 23) in the control group received radioiodine at a dose of 4.5 mCi (equivalent to 16 MBq).
Study design:	Retrospective case-control study.
Outcome Studied:	Total thyroxine (TT4) concentration, thyroid stimulating hormone (TSH), creatinine, urine specific gravity (USG) was measured before treatment and at 1, 3 and 6 months post-treatment.
Main Findings (relevant to PICO question):	At 6 months after treatment, 31/57 (54%) cats were euthyroid and 11/23 (48%) control cats were euthyroid. At 6 months after treatment, 17/57 (30%) cats were hypothyroid and 10/23 (43%) control cats were hypothyroid. At 6 months after treatment, 9/57 (16%) cats were hyperthyroid and 2/23 (9%) control cats were hyperthyroid. Combining both groups, cats that remained hyperthyroid following treatment had the highest initial serum T4 concentrations. Cats that become hypothyroid had the lowest initial serum T4 concentrations. There was no significance in these differences however. Most cats becoming euthyroid after treatment were found to have asymmetrical bilateral or unilateral disease.
Limitations:	Retrospective studies are relatively low down on the evidence hierarchy. Longer-term evaluation of thyroid parameters would provide a better idea of continued euthyroidism or relapse to hyperthyroidism. Relatively small sample size. Referral population.

**Peterson et al. (2018):** 

Population:	Client-owned cats with confirmed hyperthyroidism referred for radioiodine therapy.
Sample size:	262 cats.
Intervention details:	Cats were treated with radioiodine (1.2–13.9 mCi equivalent to 45–515 MBq).
Study design:	Prospective cohort study.
Outcome Studied:	Pre- and post-radioiodine treatment (4–8 months) measurement of: Body condition and muscle condition scores. Creatinine, urea nitrogen, SDMA concentration. Serum total thyroxine (TT4) concentration. Thyroid stimulating hormone (TSH) concentration.
Main Findings (relevant to PICO question):	209/262 (79.8%) cats became euthyroid (serum T4 and TSH concentration within the reference interval). 53/262 (20.2%) cats developed iatrogenic hypothyroidism (5 had overt hypothyroidism and 48 had subclinical hypothyroidism).
Limitations:	Cats with persistent hyperthyroidism were excluded from the study after 3 months. The wash out period for antithyroid drugs prior to radioiodine treatment was a minimum of 7 days. Referral population.

**Volckaert et al. (2018):** 

Population:	Client-owned cats with confirmed hyperthyroidism (clinical signs, increased serum T4 and scintigraphic findings) referred for radioiodine therapy.
Sample size:	75 cats.
Intervention details:	Cats were treated with radioiodine (74–259 MBq).
Study design:	Retrospective case-control study.
Outcome Studied:	Outcome of radioiodine therapy measured after 6 months: Total thyroxine (T4) concentration. Clinical signs and clinical examination.
Main Findings (relevant to PICO question):	39/75 (52%) cats were euthyroid at 6 months after radioiodine therapy. 11/75 (15%) cats were persistently hyperthyroid at 6 months after radioiodine therapy. 25/75 (33%) cats had a total T4 concentration below the reference interval at 6 months after radioiodine therapy. Outcome of radioiodine therapy is likely influenced by a multitude of factors including thyroid volume, multifocal disease, pre-therapy T4 concentration and pre-treatment antithyroid medication.
Limitations:	Retrospective studies are relatively low down on the evidence hierarchy. Referral population.

**Fernandez et al. (2019):** 

Population:	Client-owned cats with confirmed hyperthyroidism referred for radioiodine therapy.
Sample size:	55 cats.
Intervention details:	Cats were treated with radioiodine (2.0–3.4 mCi, equivalent to 74–126 MBq).
Study design:	Cohort study.
Outcome Studied:	Outcome of radioiodine therapy measured after 6–9 months: Serum thyroxine (T4) concentration. Thyroid stimulating hormone (TSH) concentration. Blood pressure. Full haematology and biochemistry. Urinalysis.
Main Findings (relevant to PICO question):	44/55 (80%) cats were euthyroid prior to radioiodine treatment owing to thyroid stabilisation with anti-thyroid medication and iodine-restricted diets. Cats were likely more sensitive to the effects of radio-iodine after ceasing anti-thyroid medication. Serum T4 concentration within reference interval in 14/55 (25.5%) cats 19 days after radioiodine treatment. Serum T4 concentration was decreased in 33/55 (60%) cats and increased in 6/55 (10.9%) cats 19 days following radioiodine treatment. 4/55 (7.3%) cats remained persistently hyperthyroid 6–9 months post-radioiodine. 22/55 (40%) cats were euthyroid 6–9 months post- radioiodine. 22/55 (40%) cats were overtly hypothyroid 6–9 months post-radioiodine. 7/55 (12.7%) cats were subclinically hypothyroid 6–9 months post-radioiodine.
Limitations:	Small population size. Referral population.

**Finch et al. (2019):** 

Population:	Client-owned cats with confirmed hyperthyroidism referred for radioiodine treatment.
Sample size:	45 cats.
Intervention details:	Cats were treated with low-dose radioiodine (111 MBq): 25 cats completed 1 and 6 month follow-up visit. 20 cats completed 12 month follow-up visit.
Study design:	Cohort study with longitudinal follow-up.
Outcome Studied:	Objective assessment of: Total thyroxine T4 (TT4) concentration and thyroid stimulating hormone (TSH) concentration. Hypothyroidism assessed as TT4 < 15 nmol/L, TSH > 0.15 ng/mL. Subclinical hypothyroidism assessed as TT4 15–60 nmol/L, TSH > 0.15 ng/mL. Euthyroidism assessed as TT4 < 60 nmol/L, TSH < 0.15 ng/mL. Hyperthyroidism assessed as TT4 > 60 nmol/L, TSH < 0.03 ng/mL. The reference boundaries for each group were based on a study by Wakeling et al. (2011). Glomerular filtration rate: serum creatinine and slope-intercept iohexol clearance.
Main Findings (relevant to PICO question):	14/20 (70%) cats were euthyroid 12-months after radioiodine treatment. Only 2/20 (10%) were persistently hyperthyroid over 12 months and required a second radioiodine treatment. 5/20 (25%) cats were hyperthyroid within 1 month of treatment but were euthyroid by 6 months. 4/20 (20%) cats developed overt or subclinical hypothyroidism within 12 months after radioiodine treatment.
Limitations:	Small study power reduces the power of the evidence. The study only included cats with mild-moderate hyperthyroidism and as such, they only received a low-dose of radioiodine therapy (111 MBq). Referral population.

**Yi et al. (2019):** 

Population:	Client-owned cats with confirmed hyperthyroidism referred for radioiodine treatment.
Sample size:	15 cats.
Intervention details:	Cats were treated with radioiodine (100–200 MBq).
Study design:	Cohort study.
Outcome Studied:	Objective assessment 7 and 14 days after radioiodine treatment: Serum thyroxine (T4) concentration. Haematology and biochemistry. Coagulation panel: fibrinogen, partial thromboplastin time (PT) and activated partial thromboplastin time (aPTT), thromboelastography.
Main Findings (relevant to PICO question):	Median T4 concentration significantly decreased in hyperthyroid cats 7 days and 14 days after radioiodine treatment. After 14 days, 7/15 cats (46.7%) had normal T4 and 8/15 (53.3%) had low T4.
Limitations:	T4 was only followed up over 14 days following radioiodine administration. Small population size reduces the power of the evidence. Since TSH was not evaluated 1–3 months after treatment as per the guidelines, euthyroid cats could not be distinguished from subclinical or overtly hypothyroid cats. The terms ‘low T4’ and ‘normal T4’ were used. Although concurrent disease was an exclusion criterion for the study population, radiation-induced thyroiditis could influence thyroid hormone levels in the short-term. An extended study would be needed to determine absolute T4 levels in response to radioiodine treatment. Referral population.

**Yu et al. (2019):** 

Population:	Client-owned cats with confirmed hyperthyroidism referred for oral radioiodine treatment.
Sample size:	161 cats.
Intervention details:	Cats were treated with a single dose of oral radioiodine (3.7 mCi equivalent to 137 MBq).
Study design:	Retrospective case-control study.
Outcome Studied:	Objective measurement of: Total thyroxine T4 concentration. Serum creatinine concentration and urinalysis, where available. Samples measured pre-radioiodine and at least 1 month after.
Main Findings (relevant to PICO question):	At re-check, 133/161 (82.6%) cats had a total T4 concentration within the reference interval. At re-check, 4/161 (2.5%) cats had a total T4 concentration above the reference interval – persistent hyperthyroidism. At re-check, 24/161 (14.9%) cats had a total T4 concentration below the reference interval.
Limitations:	Samples across the study population were analysed in different laboratories with different reference ranges. Only T4 concentration was used as a marker the severity of hyperthyroidism without considering other factors including goitre size or clinical signs. The duration between diagnosis to radioiodine treatment and duration between radioiodine and follow-up varied substanially between cats. It may be possible that relapse into hyperthyroidism or longer-term thyroid changes were missed or inconsistent. Retrospective studies are relatively low down on the evidence hierarchy. Referral population.

**Kongtasai et al. (2021):** 

Population:	Client-owned cats with confirmed hyperthyroidism referred for radioiodine treatment.
Sample size:	Total cats = 109: Hyperthyroid cats = 49 Healthy control cats = 45 Chronic kidney disease = 9 Cats with concurrent CKD and hyperthyroidism at time of diagnosis were excluded (n = 6).
Intervention details:	Cats were treated with a single dose of radioiodine (67.7–455.1 MBq; n = 49).
Study design:	Retrospective cross-sectional study for the healthy control group. Longitudinal study for the cats treated with radioiodine.
Outcome Studied:	Objective measurement of: Total thyroxine T4 concentration and thyroid stimulating hormone (TSH) levels were measured at diagnosis (T0), then 1 month after radioiodine treatment (T1) and again 11–29 months after radioiodine treatment (T2). Hyperthyroidism was diagnosed based on compatible clinical signs, increased serum T4 levels or thyroid uptake of pertechnetate. Serum creatinine concentration and urinalysis, where available. Thoracic radiographs and abdominal ultrasound to identify concurrent disease. Complete blood count (CBC).
Main Findings (relevant to PICO question):	49/49 cats had T4 levels recorded at T0 and T1. 26/49 cats had T4 levels recorded at T2 (23/49 cats were lost to follow-up due to owner non-compliance or death before assessment). At T0 all cats were hyperthyroid. At T1, 41/49 (84%) cats were euthyroid, 4/49 (8%) cats were sub clinically hypothyroid, 1/49 (2%) cat was hypothyroid, 1/49 (2%) cat remained hyperthyroid and 2/49 (4%) cats had uncertain thyroid status because they were missing TSH values. At T2, there were 18/26 (69%) euthyroid cats, 7/26 (27%) sub clinically hypothyroid cats and 1/26 (4%) iatrogenic hyperthyroid cat (levothyroxine overdose). 2/26 (8%) cats developed post-treatment azotaemia at T2.
Limitations:	23/49 cats were lost to follow-up at T2 due to owner non-compliance (n = 21) or death (n = 2). T2 varied greatly between 11–29 months. Referral population.

**Peterson & Rishniw (2021a):** 

Population:	Client-owned cats with confirmed hyperthyroidism referred for radioiodine treatment. Excluded cats with azotaemia.
Sample size:	1400 cats with hyperthyroidism.
Intervention details:	Cats were treated with a single dose of individualised radioiodine that was calculated taking into account patient-specific dosing variables.Cats with mild disease received < 66.6 MBq.Cats with moderate disease received 66.6–92.5 MBq. Cats with severe disease received > 92.5 MBq.
Study design:	Prospective case series (before and after study).
Outcome Studied:	Objective measurement of: Complete blood count (CBC), biochemistry and urinalysis. Total thyroxine T4 concentration. T3 concentration. Thyroid stimulating hormone (TSH) concentration. Qualitative and quantitative thyroid scintigraphy.
Main Findings (relevant to PICO question):	1047/1400 (74.8%) cats became euthyroid. 57/1400 (4.1%) cats became overtly hypothyroid. 240/1400 (17.1%) became sub-clinically hypothyroid. 56/1400 (4%) cats remained hyperthyroid. Cats with sub-clinical and overt hypothyroidism were older than euthyroid cats. Cats with persistent hyperthyroidism were younger than euthyroid cats. Hyperthyroid cats with bilateral disease were 1.5 times more likely to develop radioiodine-induced hypothyroidism than cats with unilateral nodules.
Limitations:	Referral population. No comparison made with other radioiodine protocols.

**Peterson & Rishniw (2021b):** 

Population:	Client-owned cats with confirmed hyperthyroidism referred for radioiodine treatment.
Sample size:	1400 cats with hyperthyroidism.
Intervention details:	Cats were treated with a single dose of radioiodine (67.7–455.1 MBq).
Study design:	Prospective case series (before and after study).
Outcome Studied:	Objective measurement of: Complete blood count (CBC), biochemistry and urinalysis. Total thyroxine T4 concentration. Triiodothyronine (T3) concentration. TSH concentration. Qualitative and quantitative thyroid scintigraphy Measurements taken before and 6–12 months after radioiodine therapy. Cats were treated with a single dose of individualised radioiodine that was calculated taking into account patient-specific dosing variables.Cats with mild received < 66.6 MBq.Cats with moderate disease received 66.6–92.5 MBq.Cats with severe disease received > 92.5 MBq.
Main Findings (relevant to PICO question):	1047/1400 (74.8%) cats become euthyroid 6–12 months following radioiodine therapy. 57/1400 (4.1%) cats become overtly hypothyroid 6–12 months following radioiodine therapy. 240/1400 (17.1%) cats become sub-clinically hypothyroid 6–12 months following radioiodine therapy. 56/1400 (4%) cats remained persistently hyperthyroid 6–12 months following radioiodine therapy. More overtly 41/57 (71.9%) and sub clinically 95/240 (39.6%) hypothyroid cats became azotaemic compared to euthyroid cats 149/1047 (14.2%).
Limitations:	Referral population. No comparison made between other radioiodine protocols. Underestimation of azotaemic cats was possible as muscle wastage may cause a falsely low serum creatinine.

## Appraisal, application and reflection

### Summary

Hyperthyroidism is the most common endocrinopathy diagnosed in senior and geriatric cats with a prevalence of 8.7% in cats >10 years of age reported in the UK (Stevens et al., 2014). Management of the disease must be considered on an individual basis considering a multitude of factors including but not limited to the underlying thyroid pathology, concurrent disease especially chronic kidney disease, severity and duration of disease, response to previous treatments, owner finances, available facilities and cat compliance. Radioiodine therapy and thyroidectomy provide definitive therapies for hyperthyroidism.

Many studies that were appraised defined the response to radioiodine therapy and thyroidectomy by the proportion of cats with thyroxine (T4) values within normal reference interval. Many of the studies do not include thyroid stimulating hormone (TSH) analysis so subclinical hypothyroidism could be missed in some cats. Currently, no assay is available that has the sensitivity to detect low TSH levels in cats, but it is possible that a proportion of cats with normal T4 levels may have subclinical hyperthyroidism (normal T4 levels but low TSH levels). This was a limitation that should be considered in answering the PICO and designing future studies.

Post-procedure follow-up of thyroid parameters is a relatively new component in the long-term management and study of feline hyperthyroidism. Generally, more recent papers had incorporated the follow-up period into their study design. The follow-up times varied following radioiodine therapy and thyroidectomy from the hours post-therapy to longer-term follow-ups over years. Peterson et al. (1995) showed that 81.3% cats (422/519) had normal T4 levels 2–3 months following radioiodine therapy, increasing to 87.1% cats (437/502) 6–12 months after radioiodine therapy. When considering the PICO, the length of follow-up should be considered and more prospective studies are needed to clarify the definitive effect of either radioiodine or thyroidectomy on T4 levels in extended follow-up periods.

One limitation across most of the papers reported is that the study populations were identified from referral level practice. It is possible that referral populations may be less reliable in predicting behaviours in primary care populations. Referral practices offer more technical diagnostics and therapies, and often treat patients with more complicated hyperthyroidism. Further studies are needed to look at the long-term outcome of cats managed in primary care setting, especially since thyroidectomy is offered in many practices when radioiodine is not an option for owners. It may be beneficial to also include qualitative studies to understand owner perspectives, concerns and commitment in managing hyperthyroidism long-term.

### Radioiodine Therapy

There was a considerably higher number of publications about the follow-up period to radioiodine therapy in cats with hyperthyroidism, compared to surgery. These studies were mainly cohort studies and the majority were prospective in nature, strengthening the evidence of their conclusions. Of 12 papers reporting T4 levels 6 months after radioiodine therapy, approximately 40–87% cats had normal T4.

There was only one randomised controlled trial. In this approximately 85% of cats were euthyroid up to 4 years following treatment (Théon et al., 1994). Peterson et al. (2018) demonstrated in a prospective cohort study that 79.8% cats (209/262) were euthyroid (normal T4 and TSH) 6 months following radioiodine therapy. Although this study distinguished between euthyroid and sub-clinically hypothyroid cats, persistently hyperthyroid cats were excluded from their statistics and longer-term T4 levels were not reported. There were numerous retrospective studies, which by nature provide weaker evidence supporting the PICO. However, many of these retrospective studies had strict inclusion criteria, large population sizes and the power to follow-up longer-term responses. A retrospective study by Chun et al. (2002) in a study population of 193 cats found that over 150 had normal T4 levels 12 months after radioiodine therapy, or 77.7% (a similar finding to the prospective study by Peterson et al., 2018). Finch et al. (2019) found that five cats that were hyperthyroid 1 month after radioiodine therapy were euthyroid by 6 months in a relatively small population of 25 cats. A limitation of most of these papers, especially the retrospective studies, was that they were mainly testing other variables including radioiodine dosing or pre-radioiodine T4 stabilisation.

As discussed previously, more prospective data is needed throughout the follow-up period following radioiodine therapy, with controls including using the same testing laboratory for T4 values, to identify the true proportion of cats that have normal T4, iatrogenic hypothyroidism or persistent hyperthyroidism after treatment.

### Thyroidectomy

Only five publications were evaluated exploring how thyroidectomy influenced long-term T4 levels after surgery, specifying euthyroid status. Three of these papers were published prior to 2000 where long-term T4 monitoring was not incorporated into study objectives.

The most recent publication by Covey et al. (2019) reported 13/68 (19%) were euthyroid 6 months after bilateral thyroidectomy and 19/23 (83%) were transiently euthyroid 595–1955 days after bilateral thyroidectomy; a value similar to the proportion of cats with normal T4 levels following radioiodine therapy. Only 23 cats were analysed long term in this retrospective study, which by nature is a weaker form of evidence. The study reported a high proportion of recurrent hyperthyroidism 10/23 (44%) in cats after surgery and over time cats were more likely to revert from euthyroidism to hyperthyroidism. Although a smaller population size, the study measures TSH to confidently distinguish between euthyroidism and hypothyroidism. The remaining four studies measured T4 levels in cats receiving both unilateral and bilateral thyroidectomy. DiBartola et al. (1996) found that T4 levels were significantly lower following bilateral thyroidectomy but did not report the normal reference interval.

Interestingly they did not find a significant difference between T4 levels between day 30 and day 90 post-surgery. There was also no significant difference between surgery and radioiodine therapy in this study. Graves et al. (1994) found that 46% (6/13) cats were euthyroid following bilateral thyroidectomy and the remaining were hypothyroid 30 days after surgery. Birchard et al. (1984) followed 85 cats prospectively, which increases the strength of the evidence. There were relatively small numbers of cats in each group; 32 receiving unilateral thyroidectomy and 53 receiving bilateral thyroidectomy. It was found that all cats had T4 within the reference interval 48 hours following surgery but four cats from the bilateral thyroidectomy group relapsed into hyperthyroidism within 8–44 months after surgery. Although the exact number was not included, the study reported that the majority of cats had below normal T4 2–3 months after unilateral thyroidectomy but did not require T4 supplementation since cats did not show outward clinical signs of hypothyroidism. The importance of long-term thyroid evaluation is important following either unilateral or bilateral thyroidectomy to identify subclinical hypothyroidism and hyperthyroid remission.

Naan et al. (2006) found that all cats that had undergone unilateral thyroidectomy were in remission long-term. True unilateral disease was confirmed with thyroid imaging and as such, unilateral thyroidectomy was very successful on long-term thyroid evaluation. Only nine cats had true unilateral disease and the study was retrospective. In bilateral disease, many cats have asymmetrical thyroid enlargement with one lobe being very enlarged and one lobe minimally enlarged. Unilateral thyroidectomy may restore euthyroidism in these cats and it could take many months before the remaining lobe grows enough for hyperthyroidism to recur (Peterson, 2011). A prospective study with more cats may be difficult to perform since radioiodine is more widely available and associated with higher remission and fewer clinical signs. The 81 cats that received bilateral thyroidectomy remained in remission 13 months following surgery.

A large proportion of studies did not measure TSH levels, alongside T4 levels, in cats following either thyroidectomy or radioiodine therapy. Without TSH levels, it is difficult to distinguish between euthyroidism and subclinical hypothyroidism following treatment. Finch et al. (2019) measures T4 and TSH levels and found that 20% cats developed overt or subclinical hypothyroidism within 12 months of radioiodine therapy.

### Conclusion

In answer to the PICO, there is more evidence available from observational studies showing that radioiodine therapy is more effective at normalising T4 levels compared to unilateral or bilateral thyroidectomy. It is relatively new for studies to incorporate long-term T4 monitoring in their study designs, particularly focusing on hyperthyroidism remission and iatrogenic hypothyroidism. More studies are required with a prospective or randomised design with larger sample sizes and lifelong thyroid evaluation to improve the evidence-base available. This is especially important in determining the long-term effect of thyroidectomy on T4 values, as fewer papers are available.

## Methodology Section

**Search Strategy:** 

Databases searched and dates covered:	CAB Abstracts on OVID Platform 1973 to Week 16 2022PubMed NCBI 1964 to August 2021
Search strategy:	CAB Abstracts: (cat or cats or feline or felines or felis).mp. or exp cats/ or exp felis/ (hyperthyroid or hyperthyroidism).mp. or exp hyperthyroidism/ (I-131 or radioiodine or radioactive iodine).mp. (thyroidectomy or thyroidectomies or (thyroid and surg*)).mp. or exp thyroidectomy/ Search:1 and 2 and (3 or 4) PubMed: (cat or cats or feline or felines or felis) (hyperthyroid or hyperthyroidism) (I-131 or radioiodine or radioactive iodine) (thyroidectomy or thyroidectomies or (thyroid and (surgery or surgical)) Search: #1 and #2 and (#3 or #4)
Dates searches performed:	28 Apr 2022

**Exclusion / Inclusion Criteria:** 

Exclusion:	Unrelated to the PICO, single case reports, book chapters, conference proceedings, reviews, articles not in English, studies that did not evaluate T4 levels at a suitable pre and post-intervention interval, studies that did not define the proportion of cats that were euthyroid or normal T4 reference range after radioiodine, studies using cats that have received both thyroidectomy and radioiodine in their clinical history.
Inclusion:	Related to the PICO, journals in English, studies that made comparison between T4 level pre and post-intervention.

**Search Outcome:** 

Database	Number of results	Excluded – Relevance	Excluded – Language	Excluded – Duplicates	Excluded – No Access	Total relevant papers
CAB Abstracts	309	265	9	1	1	33
PubMed	175	151	2	0	0	22
Total relevant papers when duplicates removed						35

## ORCID

Alexander M. Davies: https://orcid.org/0000-0001-6828-0430

## Conflict of Interest

The author declares no conflicts of interest.
